# Editorial

**Published:** 1838-01-17

**Authors:** 


MEDICAL EXAMINER.

PHILADELPHIA.

Wednesday, January 17, 1838.

We regret, that the number and length of the clinical lectures oblige us to omit several original communications, editorial matter, and many items of foreign and domestic intelligence of interest. In addition to our usual sixteen pages, we issue to day an extra sheet ef 8 pages.

After to day, the Examiner will not be sent to subscribers out of the city, unless the amount of the subscription has been paid.

